# Alterations in the connection topology of brain structural networks in Internet gaming addiction

**DOI:** 10.1038/s41598-018-33324-y

**Published:** 2018-10-11

**Authors:** Chang-hyun Park, Ji-Won Chun, Hyun Cho, Dai-Jin Kim

**Affiliations:** 1 Department of Psychiatry, Seoul St. Mary’s Hospital, College of Medicine, Catholic University of Korea, Seoul, Korea; 2 Department of Psychology, Korea University, Seoul, Korea

## Abstract

Internet gaming addiction (IGA), as the most popular subtype of Internet addiction, is becoming a common and widespread mental health concern, but there are still debates on whether IGA constitutes a psychiatric disorder. The view on the brain as a complex network has developed network analysis of neuroimaging data, revealing that abnormalities of brain functional and structural systems are related to alterations in brain network configuration, such as small-world topology, in neuropsychiatric disorders. Here we applied network analysis to diffusion-weighted MRI data of 102 gaming individuals and 41 non-gaming healthy individuals to seek changes in the small-world topology of brain structural networks in IGA. The connection topology of brain structural networks shifted to the direction of random topology in the gaming individuals, irrespective of whether they were diagnosed with Internet gaming disorder. Furthermore, when we simulated targeted or untargeted attacks on nodes, the connection topology of the gaming individuals’ brain structural networks under no attacks was comparable to that of the non-gaming healthy individuals’ brain structural networks under targeted attacks. Alterations in connection topology provide a clue that Internet gaming addicted brains could be as abnormal as brains suffering from targeted damage.

## Introduction

Internet gaming addiction (IGA) is characterized primarily by excessive or compulsive use of Internet-based games. Although research on IGA is increasingly growing, the research is still in the early stage and thus conducive to controversy over its causes^1^. Indeed, the fifth edition of the Diagnostic and Statistical Manual of Mental Disorders (DSM-5)^2^ concluded that there was insufficient evidence to include IGA as an official mental disorder, such that it is in the status of “Conditions for Further Study” in the DSM-5.

Aside from critiques on the causes of IGA, brain functional and structural changes related to IGA have been revealed in a large volume of literature (for review, see (Weinstein^3^). In addition to local-scale changes in Internet gaming addicted brains, whole brain-scale changes were also sought by modelling the brain as a network consisting of functional or structural connections between regions and then by measuring properties of the network. But changes at the whole brain scale appear to be unclear or inconsistent in both functional^4,5^ and structural^6,7^ networks of Internet gaming addicted brains.

Small-world topology^8^, which involves intermediate properties between regular and random topology, is among main features of complex networks such as the brain as a network. It has been revealed that functional and structural networks of healthy brains exhibit small-world topology, and furthermore, the connection topology in brains with neuropsychiatric disorders could be away from the healthy brains’ small-world topology to the direction of regular or random topology^9^. The connection topology of a network can be assessed by computing network measures: for instance, in terms of global and local efficiency^10^ that reflect the efficiency in transporting information at global and local scales respectively, a network of small-world topology, that is, a small-world network has greater global efficiency than a regular network as well as greater local efficiency than a random network. In our previous study^11^, we proposed that the connection topology of brain functional networks could change towards random configuration in IGA by exhibiting increased global efficiency and decreased local efficiency in gaming individuals.

In this investigation, we were primarily concerned with whether such whole brain-scale changes could be seen for brain structural networks as well in IGA. To be more specific, firstly, we were interested in whether topological changes of brain structural networks would be exhibited only in gaming individuals diagnosed with Internet gaming disorder (IGD) or even in those diagnosed as not being IGD. Secondly, we wondered whether similar topological changes could be seen when we simulated targeted or untargeted attacks on brain structural networks. Thirdly, we wanted to assess the predictive value of topological changes in discriminating gaming individuals from non-gaming healthy individuals.

## Methods

### Participants

Among individuals playing Internet-based games, 102 individuals (25.85 ± 4.69 years; 40 females) without any comorbidity were included in the study. The diagnosis of IGD in them was made by employing nine criteria for characterizing IGD as provided in the DSM-5. Among the 102 gaming individuals, 51 individuals (27.08 ± 5.19 years; 19 females) who satisfied at least five criteria were labelled IGD and the other 51 individuals (24.63 ± 3.81 years; 21 females) who satisfied less than five criteria were labelled IGC (Internet gaming control). The diagnosis according to the nine criteria was verified by a structured interview with a clinical psychologist, so that any individual who showed a mismatch between the two was excluded. In addition, 41 healthy individuals (25.46 ± 3.94 years; 15 females) not playing Internet-based games were separately recruited and they were labelled NHC (non-gaming healthy control). Written informed consent was obtained from all participants in accordance with the Declaration of Helsinki and its later amendments, and the study was approved by the Institutional Review Board at the Seoul St. Mary’s Hospital, Seoul, Korea.

### Acquisition and preprocessing of dMRI data

Diffusion-weighted MRI (dMRI) data were obtained using a MAGNETOM Verio 3 T system (Siemens AG, Erlangen, Germany). For each participant, 31 volume images were acquired with a single-shot diffusion-weighted echo planar imaging sequence. The data set consisted of 30 volume images with high diffusion weighting (*b* value = 1,000 s/mm^2^) applied along respective diffusion gradient encoding directions and one volume image with no diffusion weighting (*b* value = 0 s/mm^2^). Each volume image included 2.0 mm thick 75 axial slices of a 114 × 114 matrix with 2.0 mm × 2.0 mm in-plane resolution.

The acquired raw dMRI data was preprocessed using the tools in FSL 5.0.9 (http://fsl.fmrib.ox.ac.uk/fsl/). Each participant’s 31 volume images were realigned to the volume image with no diffusion weighting to correct for eddy current-induced distortions and head motion, and then non-brain tissue was deleted from the whole head in the realigned volume images.

### White matter tractography on dMRI data

Using the tools in Diffusion Toolkit 0.6.4 (http://trackvis.org/dtk/), a diffusion tensor was modelled in every voxel of the preprocessed dMRI data and diffusion tensor-derived parameters including fractional anisotropy (FA)^12^ were computed. Given three diffusivities along different axes of a diffusion tensor, FA was calculated as a measure of diffusion anisotropy on the basis of diffusivity differences between the three axes. Based on the information of the diffusion tensor and diffusion tensor-derived parameters, white matter (WM) tractography was carried out to reconstruct whole brain fibre tracts by applying a deterministic tractography algorithm, specifically the interpolated streamline method, with the following options: number of seeds per voxel = 8, step length = 1.0 mm, angle threshold = 45°, and FA threshold = 0.1.

**Figure 1:**
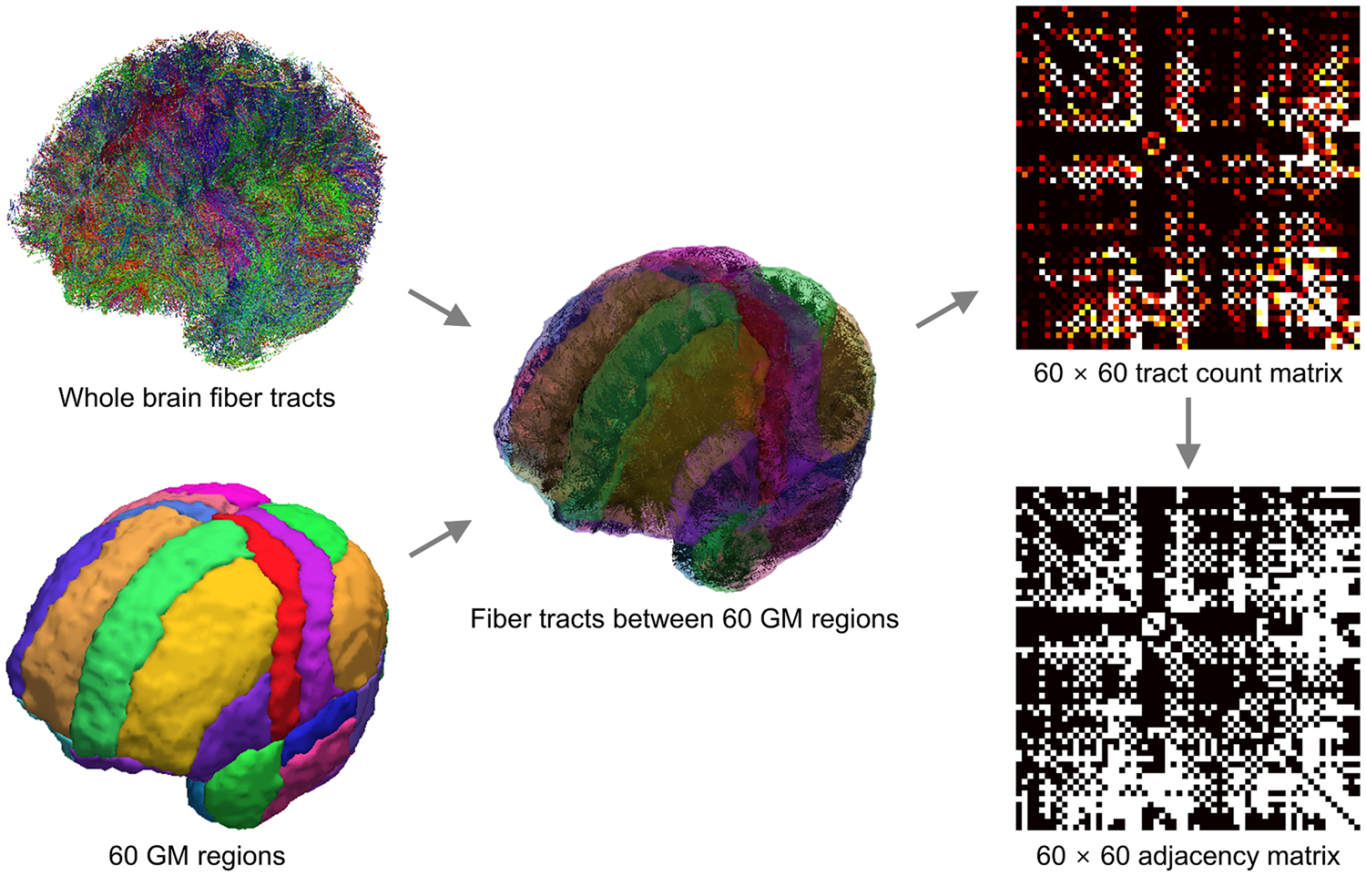
Schematic procedures for constructing brain structural networks. The number of white matter fibre tracts connecting each pair of 60 grey matter regions composed a 60 × 60 tract count matrix, which was then converted to an adjacency matrix consisting of 1’s and 0’s.

When examining the topological properties of brain networks across a number of individuals, connected networks without isolated nodes across all the individuals are often preferably considered^13^. To specify WM fibre tracts between pairwise grey matter (GM) regions, 60 regions (Supplementary Table S1) were defined on the whole brain GM, as parcellated in the Hammers atlas^14^, rather than more finely parcellated atlases that may yield unconnected networks. The 60 GM regions were transformed from the standard space on which the atlas was originally defined to the native space on which the reconstructed whole brain fibre tracts were defined. The number of WM fibre tracts that started and ended in each pair of the 60 GM regions was counted from the reconstructed whole brain fibre tracts, and a 60 × 60 matrix of tract counts was generated. The matrix of tract counts was then converted to an adjacency matrix consisting of 1’s and 0’s. The schematic procedures for acquiring the adjacency matrix is presented in Fig. 1.

### Computation of network measures

In the adjacency matrix, regions corresponded to nodes and 1’s and 0’s represented edges and anti-edges (absence of edges), respectively, between the nodes in a network. In converting the tract count matrix to the adjacency matrix, the number of 1’s, that is, the number of edges in a network was set to be the same for all individuals, since inter-individual differences in network density can influence network measures^15^. The common network density was determined as 0.4328, the lowest density at which connected networks were constructed across all individuals.

For each individual’s brain structural network, we measured global and local efficiency to specify the connection topology of the network. Global efficiency represents closeness between general nodes in the scope of an entire network, while local efficiency indicates closeness between nearby nodes in the scope of a local subnetwork of the network. When considering regular and random networks as extreme networks in terms of connection topology, global efficiency is the smallest in the regular network and the greatest in the random network, whereas local efficiency is the smallest in the random network and the greatest in the regular network. When healthy brains are shown to be a small-world network, differences in global and local efficiency from healthy brains could be regarded as representing a shift in connection topology from the small-world network towards either of extreme networks.

We also employed another network measure, omega, which summarizes the degree of being close to a small-world network in terms of connection topology. We computed omega by modifying its original suggestion^16^ in such a way that global and local efficiency was compared to that of equivalent extreme networks: the ratio of global efficiency of a given network to that of the equivalent random network minus the ratio of local efficiency of a given network to that of the equivalent regular network. Omega accordingly ranged between −1 and 1 that corresponded to regular and random networks, respectively, as extreme networks, and it was close to zero for a small-world network. Given a network, regular and random networks were generated 100 times by randomizing and latticizing the network while preserving its degree distribution, so that omega was computed as the average across the 100 realizations of the extreme networks.

The formal mathematical definitions of the network measures adopted in this study are provided in Supplementary Methods. For the computation of the network measures, we used functions in the Brain Connectivity Toolbox (https://sites.google.com/site/bctnet/) and in-house functions that implemented the definitions.

### Classification performance of network measures

Logistic regression and support vector machines (SVMs) were employed to construct models for classifying between each subgroup of the gaming individuals and NHC and also between IGD and IGC. Values of global and local efficiency were used as predictors of the classifiers. For the SVMs, a linear kernel was used as the kernel function and hyperparameters were tuned via five-fold cross-validation (CV). We assessed accuracy in the classification between each pair of the three groups via leave-one-out CV (LOOCV). That is, out-of-sample classification accuracy was computed for each left-out individual, and then it was averaged across all individuals.

The statistical significance of classification accuracy was estimated by employing permutation testing. In the classification between each pair of the three groups, classification accuracy provided via LOOCV for the original labels of the individuals was compared to an empirical null distribution that was generated by repeatedly permuting the original labels and computing classification accuracy via LOOCV for the permuted labels 10,000 times. If classification accuracy obtained for the original labels was equal to or higher than that observed for the permuted labels at the significance level of a *p* value of 0.05, the achieved accuracy in the classification between the two groups was determined to be significantly different from the chance level (50%).

### Simulation of network attacks

For each individual’s brain structural network, attacks on nodes were simulated in two different ways^17^. In one way, targeted attacks were addressed by eliminating central nodes from the original network. To assess the degree of being central of each node in the original network, node betweenness centrality^18^ was computed, which is defined as the fraction of shortest paths that pass through a given node. Having achieved targeted attacks by removing nodes of greater node betweenness centrality, network measures were computed for the attacked network. In the other way, untargeted attacks were simulated by removing randomly selected nodes from the original network. Random selection of nodes was repeated 100 times, so that network measures of the attacked network were computed as the average across the 100 realizations of the untargeted attacks.

To examine changes in connection topology according to the extent of attacks, we repeatedly simulated the two ways of attacks on 5% and 10% of all nodes. In attacks on 5% (resp. 10%) of nodes, 60 × 0.05 = 3 (resp. 60 × 0.10 = 6) nodes were determined according to their being higher in node betweenness centrality and then eliminated for targeted attacks or they were randomly selected and then removed for untargeted attacks.

### Statistical inferences

Global efficiency, local efficiency, and omega of brain structural networks were compared between each subgroup of the gaming individuals and NHC and between IGD and IGC using independent two samples Student’s *t*-tests. The comparisons were repeatedly conducted for unattacked brain structural networks and for targetedly and untargetedly attacked brain structural networks. In addition, the interaction between groups (IGD, IGC, and NHC) and attack extents (unattacked, 5% attacked, and 10% attacked) in omega was assessed for each of the targeted and untargeted attacks using repeated measures ANOVAs. For the statistical inferences, we used functions in the Statistics and Machine Learning Toolbox in MATLAB (MathWorks, Natick, Massachusetts, USA). Statistical significance was identified primarily when a *p*-value was less than 0.05 corrected for multiple comparisons using the Tukey–Kramer method^19^ and secondarily when a *p*-value was less than 0.05 but failed to pass multiple comparison correction.

## Results

### Comparison of network measures under no network attacks

**Figure 2:**
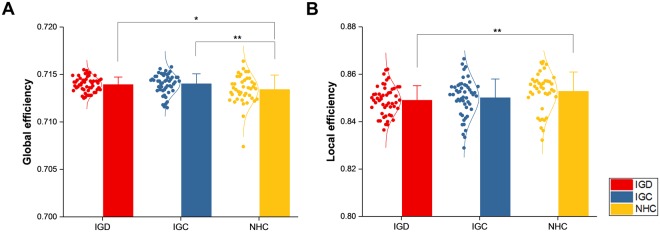
Comparison of global (**A**) and local (**B**) efficiency. The network measures were compared between gaming individuals, comprising two subdivisions labelled IGD (Internet gaming disorder) and IGC (Internet gaming control), and non-gaming healthy controls labelled NHC. Values of the network measures are represented as dots, along with a normal distribution curve fitted to them. The mean and standard deviation of the values in each group are indicated by a bar and an error bar, respectively. Single and double asterisks signify statistical significance uncorrected and corrected, respectively, for multiple comparisons.

**Figure 3:**
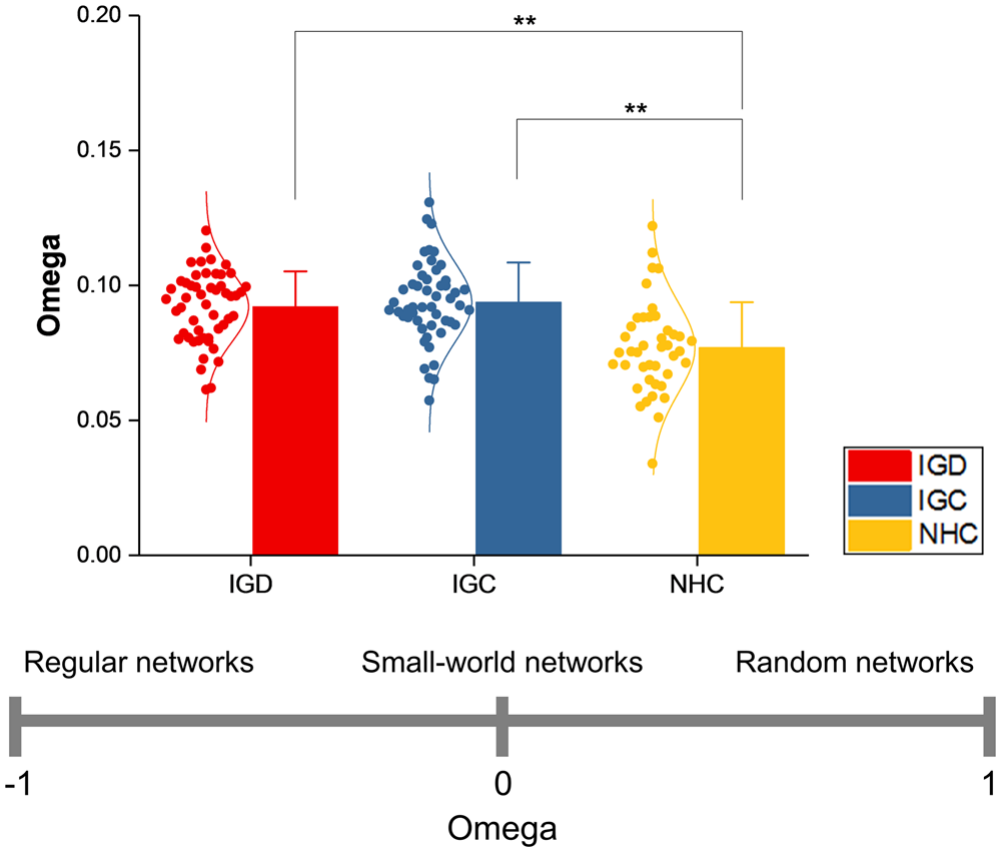
Comparison of omega. The network measure was compared between gaming individuals, comprising two subdivisions labelled IGD (Internet gaming disorder) and IGC (Internet gaming control), and non-gaming healthy controls labelled NHC. Values of the network measure are represented as dots, along with a normal distribution curve fitted to them. The mean and standard deviation of the values in each group are indicated by a bar and an error bar, respectively. Double asterisks signify statistical significance corrected for multiple comparisons. The bottom plot shows the available range of omega, corresponding to different network configuration.

In the comparison of network measures between each pair of the three groups, global efficiency was higher in IGD and IGC than in NHC (IGD vs. NHC: *t*(90) = 2.1539, *p* = 0.0339 and IGC vs. NHC: *t*(90) = 2.2404, *p* = 0.0275) (Fig. 2A), while local efficiency was lower in IGD than in NHC (IGD vs. NHC: *t*(90) = −2.4335, *p* = 0.0169) (Fig. 2B), reflecting a shift in the connection topology of brain structural networks towards a random network in the gaming individuals. Indeed, omega was much farther in the positive direction from zero in IGD and IGC than in NHC (IGD vs. NHC: *t*(90) = 4.3801, *p* < 0.0001 and IGC vs. NHC: *t*(90) = 5.0018, *p* < 0.0001) (Fig. 3).

### Classification accuracy of network measures

In the classification models using global and local efficiency as predictors, accuracy for discriminating IGD from NHC was 66.30% by logistic regression and 64.13% by an SVM, with significant differences from the chance level (Table 1). Accuracy was not significantly higher than the chance level in distinguishing IGC from NHC (57.61% by logistic regression and 60.87% by an SVM). In particular, in separating IGD from IGC, accuracy was much poorer such that it remained near the chance level with no significant differences (50.98% by logistic regression and 51.96% by an SVM).

**Table 1:** Performance of classification models constructed based on network measures.

	IGD vs. NHC	IGC vs. NHC	IGD vs. IGC
Logistic regression	66.30%	57.61%	50.98%
	(p = 0.002)	(*p* = 0.114)	(*p* = 0.326)
SVM	64.13%	60.87%	56.86%
	(*p* = 0.021)	(*p* = 0.058)	(*p* = 0.109)

Classification was performed between each subdivision of gaming individuals, labelled IGD (Internet gaming disorder) and IGC (Internet gaming control), and non-gaming healthy controls labelled NHC and between the two subdivisions of gaming individuals. Each classifier’s performance is described by accuracy in percentage and its significance in terms of a *p* value assessed via permutation testing.

SVM, support vector machine.

### Comparison of network measures under network attacks

**Figure 4:**
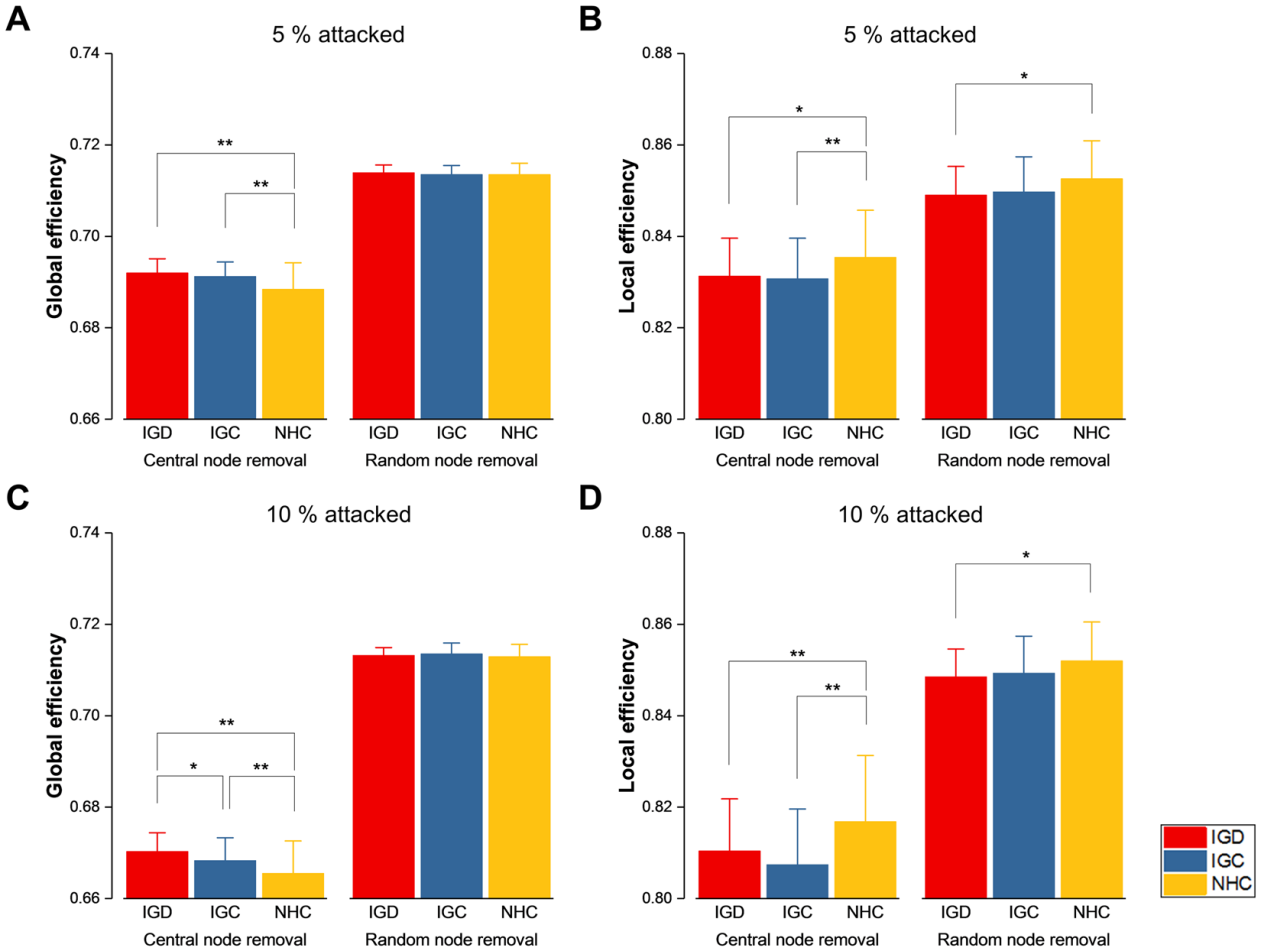
Comparison of global (**A** and **C**) and local (**B** and **D**) efficiency under network attacks. The network measures were compared between gaming individuals, comprising two subdivisions labelled IGD (Internet gaming disorder) and IGC (Internet gaming control), and non-gaming healthy controls labelled NHC. Central node removal addresses targeted attacks, and random node removal corresponds to untargeted attacks. Targeted and untargeted attacks are on 5% (**A** and **B**) and 10% (**C** and **D**) of all nodes. The mean and standard deviation of the values in each group are indicated by a bar and an error bar, respectively. Single and double asterisks signify statistical significance uncorrected and corrected, respectively, for multiple comparisons.

In the comparison of network measures between each pair of the three groups for attacked brain structural networks, global efficiency was higher (IGD vs. NHC: *t*(90) = 3.8289, *p* = 0.0002; IGC vs. NHC: *t*(90) = 2.9619, *p* = 0.0039) and local efficiency was lower (IGD vs. NHC: *t*(90) = −2.0970, *p* = 0.0388; IGC vs. NHC: *t*(90) = −2.3401, *p* = 0.0215) in IGD and IGC than in NHC against targeted attacks on 5% nodes being central in the original network. In contrast, global efficiency was not different and local efficiency was lower (IGD vs. NHC: *t*(90) = −2.3502, *p* = 0.0210) only in IGD than in NHC against untargeted attacks on 5% nodes randomly selected (Fig. 4A and B). Similar patterns of differences in the network measures were observed for attacks on 10% nodes (Fig. 4C and D). That is, increased global efficiency (IGD vs. NHC: *t*(90) = 4.1185, *p* = 0.0001; IGC vs. NHC: *t*(90) = 2.2516, *p* = 0.0268) and decreased local efficiency (IGD vs. NHC: *t*(90) = −2.3500, *p* = 0.0210; IGC vs. NHC: *t*(90) = −3.3549, *p* = 0.0012) in the gaming individuals were displayed for targeted attacks, but decreased local efficiency (IGD vs. NHC: *t*(90) = −2.2749, *p* = 0.0253) was only exhibited in IGD for untargeted attacks.

**Figure 5:**
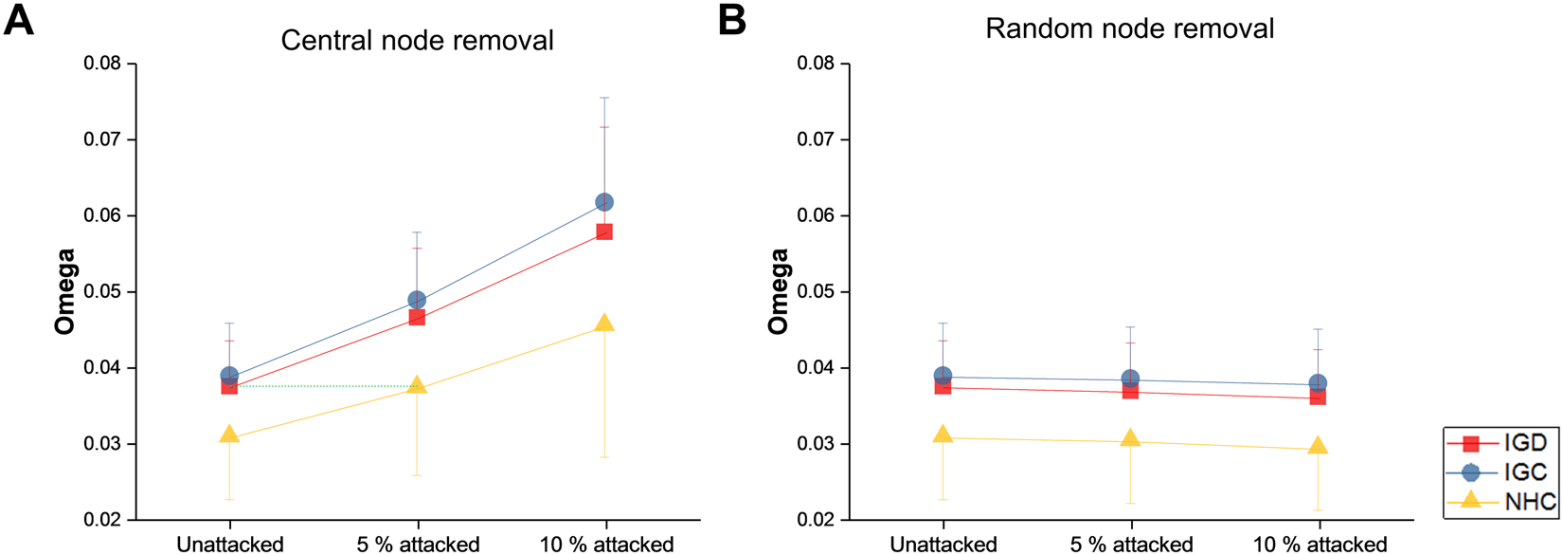
Change in omega across different extents of network attacks. The network measure was measured for gaming individuals, comprising two subdivisions labelled IGD (Internet gaming disorder) and IGC (Internet gaming control), and non-gaming healthy controls labelled NHC. Central node removal (**A**) addresses targeted attacks, and random node removal (**B**) corresponds to untargeted attacks. A square, a circle, and a triangle represent the mean and an error bar indicates the standard deviation in each group. A dotted green line shows the overlap of omega values between IGD in the unattacked state (omega = 0.0374 ± 0.006) and NHC in the 5% attacked state (omega = 0.0373 ± 0.0117).

Omega was much farther in the positive direction from zero in IGD and IGC than in NHC against targeted attacks on 5% nodes (IGD vs. NHC: *t*(90) = 4.2315, *p* = 0.0001; IGC vs. NHC: *t*(90) = 5.3288, *p* < 0.0001) and 10% nodes (IGD vs. NHC: *t*(90) = 3.7724, *p* = 0.0003; IGC vs. NHC: *t*(90) = 4.9713, *p* < 0.0001), and also against untargeted attacks on 5% nodes (IGD vs. NHC: *t*(90) = 4.2351, *p* = 0.0001; IGC vs. NHC: *t*(90) = 5.1438, *p* < 0.0001) and 10% nodes (IGD vs. NHC: *t*(90) = 4.3893, *p* < 0.0001; IGC vs. NHC: *t*(90) = 5.2703, *p* < 0.0001) (Fig. 5). But the interaction between groups (IGD, IGC, and NHC) and attack extents (unattacked, 5% attacked, and 10% attacked) in omega was seen only for targeted attacks (*F*(4, 280) = 6.2159, *p* = 0.0011 in Greenhouse-Geisser correction), indicating that differences in omega between the three groups were not consistent across the different attack extents. For targeted attacks, indeed, IGD and IGC showed a trend of steeper increases in omega compared to NHC as the extent of attacks broadened. Notably, since omega increased evidently even in NHC under targeted attacks, the omega of IGD under no attacks (omega = 0.0374 ± 0.006) overlapped with that of NHC under targeted attacks on 5% nodes (omega = 0.0373 ± 0.0117), as indicated as the green dotted line in Fig. 5A.

## Discussion

In this research, we examined whether the structural networks of Internet gaming addicted brains could show a shift in connection topology to the direction of a random network. As main findings, firstly, changes in connection topology were observed in both IGD and IGC. That is, a shift towards random topology was not limited to IGD among the gaming individuals. Secondly, targeted attacks on central nodes in brain structural networks induced a shift from small-world topology towards random topology in NHC as well as in IGD and IGC, such that unattacked brain structural networks of the gaming individuals were comparable to targetedly attacked brain structural networks of NHC in terms of connection topology.

Brain functional and structural changes induced by or related to IGA have been repeatedly observed. Specifically, functional alterations include decline in resting state functional connectivity^20–23^ and increase of activation in response to Internet gaming-related stimuli^24,25^, and structural alterations include shrinkage of GM volume^26–28^, reduction in cortical thickness^29^, and loss of WM integrity^30^. In contrast to these local-scale changes in the brain, whole brain-scale changes across the brain, particularly changes in network properties of Internet gaming addicted brains, were not clearly seen. Brain functional networks constructed from resting state functional MRI showed no alterations in network measures for IGD^4,5^ and other addictive disorders^31–33^. Moreover, brain structural networks constructed from dMRI showed no alterations^34^ or inconsistent changes^7,35^ in network measures for IGD and other addictive disorders.

Network measures may be seen as too abstract measures of the brain since the functional or structural status over the whole brain is summarized as single values^36^. In addition, network measures may have variability depending on different settings with respect to the ways of constructing networks^37–39^. In spite of these limitations, macroscopic features assessed by network measures at the whole brain scale could constitute a more comprehensive understanding of the brain. Among various properties related to brain network configuration, the connection topology of a brain network is crucial to the organization and coordination of the global states of the brain^40^, and efficient network behaviour characterized by small-world topology has been well recognized^41^.

As the virtue of topological views on brain functional and structural networks, abnormalities of brains, for instance, with neuropsychiatric disorders^9,42^, can be detected in terms of a shift in connection topology. When supposing that healthy brains have typical small-world topology in functional and structural networks, abnormal brains tend to exhibit a shift in connection topology from the small-world configuration of healthy brains. The direction of such a topological shift, which could be inferred in terms of changes in network measures such as global and local efficiency, was often shown to be towards random configuration^43,44^. In our previous study we revealed that the connection topology of brain functional networks shifted towards random configuration in IGA^11^, and in the current study we demonstrated that such a shift in connection topology towards random configuration could be seen in brain structural networks as well. These macroscopic changes at the whole-brain scale represent that Internet gaming addicted brains may have abnormalities in both function and structure.

In order to assess this notion further, we sought to examine whether similar topological changes could be seen when we simulated attacks on brain structural networks by removing nodes. We regarded node removal as restricting the roles of the nodes, and we thus wanted to connect node removal conceptually to virtual damage to GM regions corresponding to the nodes^45,46^. A shift in connection topology towards random configuration was still seen for both targeted attacks on central nodes and untargeted attacks on randomly selected nodes in the gaming individuals. Also, consistently with the notion that targeted attacks generally induce greater vulnerability compared to random attacks^46^, a shift towards a more extreme random network was primarily observed for targeted attacks even in NHC. Of note, the degree of being close to small-world topology, as assessed by omega, was similar between IGD under no attacks and NHC under targeted attacks on 5% nodes. That is, brain network configuration was comparable in terms of connection topology between gaming individuals in the unattacked state and non-gaming healthy individuals in the targetedly attacked state. Although we may not assert that Internet gaming addicted brains could be damaged brains by only relying on this topological correspondence, we suggest that alterations in brain network configuration may be a clue that Internet gaming addicted brains could be as abnormal as brains suffering from targeted damage.

From the perspective of predictive modelling, global and local efficiency may serve as neuroimaging markers for discriminating gaming individuals. When we assessed the performance of classification models composed of the just two network measures, IGD was successfully distinguished from NHC, whereas IGC was not adequately distinguished from NHC. In addition, since brain topological changes were generally comparable between IGD and IGC, classifying between the two subgroups of the gaming individuals was quite challenging. Network measures that represent brain topological alterations could contribute to the diagnosis of IGA, but we note that such whole brain-scale changes are not specific to IGA, such that it would be unlikely to be successful to classify between IGA and other neuropsychiatric disorders that exhibit similar brain topological changes.

Distinctly from other studies in which whole brain-scale changes were not clearly seen in IGA, we showed a shift in connection topology towards random configuration in our consecutive studies on brain functional and structural networks. Considering the inconsistency among them, we checked out that our findings, in particular, greater omega in the positive direction in Internet gaming addicted brains than in healthy brains, have not been affected by variations in network analysis, such as the selection of different density in constructing brain structural networks (Supplementary Fig. S1) and the adoption of different measures as the criteria for simulating targeted attacks on brain structural networks (Supplementary Fig. S2). Nevertheless, we acknowledge that the detection of such changes is only the first step for a better understanding about the brain network configuration of Internet gaming addicted brains. Furthermore, although we have focused on alterations in small-world topology in this study, we admit that small-world topology may be too limited a concept to capture the diverse topological aspects and dynamic patterns of brain networks^47^. From the network perspective of Internet gaming addicted brains, whole-brain scale changes should be thoroughly examined in forthcoming studies, not least the connection of them with local-scale changes.

In summary, in the current research, we revealed that the configuration of brain structural networks shifted to the direction of random topology in IGA. Furthermore, we showed that such brain topological alterations in Internet gaming addicted brains were comparable to those induced by virtual damage to GM regions in healthy brains. Subsequently to our previous study, alterations in connection topology imply abnormalities of Internet gaming addicted brains.

## Electronic supplementary material

Supplementary Information

## Data Availability

The datasets analysed during the current study are available from the corresponding author on reasonable request.
