# Supplementary material for: Alterations in the connection topology of brain structural networks in Internet gaming addiction
Source: Sci Rep. 2018 Oct 11;8:15117. doi: 10.1038/s41598-018-33324-y (PMC6182010; doi:10.1038/s41598-018-33324-y)
Supplement: Supplementary file 1 — Supplementary Information [file 41598_2018_33324_MOESM1_ESM.pdf]

Supplementary Information:

Alterations in the connection topology of brain structural networks in Internet gaming addiction

Chang-hyun Park[1], Ji-Won Chun[1], Hyun Cho[1,2], Dai-Jin Kim[1]

[1]Department of Psychiatry, Seoul St. Mary's Hospital, College of Medicine, Catholic University of Korea, Seoul, Korea
[2]Department of Psychology, Korea University, Seoul, Korea

Supplementary Methods

Mathematical concepts of network measures

Global and local efficiency are based on the common concept of efficiency that reflects how efficiently a network exchanges information[1]. The efficiency, ε, in the information transfer between a pair of nodes i and j is defined as the reciprocal of the distance, d_{ij}, between them: $\varepsilon_{ij} = 1/d_{ij}$. For an unweighted network, the distance may be specifically provided by the number of edges in the shortest path among all possible paths between the two nodes[2].

(i) The global efficiency of an unweighted network, **G**, is provided as the average of the efficiency between every pair of N nodes in the network: $E_{glob}(\mathbf{G}) = 1/(N(N\text{-}1)) \sum_{i \neq j \in \mathbf{G}} \varepsilon_{ij} = 1/(N(N\text{-}1)) \sum_{i \neq j \in \mathbf{G}} 1/d_{ij}$. Global efficiency exhibits the efficiency in transporting information at a global scale between generic nodes. Between 0 and 1, a higher value indicates the greater efficiency in global information exchange.

(ii) The local efficiency of an unweighted network, **G**, is provided as the average of the efficiency for every local subnetwork composed of the neighboring nodes of each node: $E_{loc}(\mathbf{G}) = 1/N \sum_{i \in \mathbf{G}} E(\mathbf{G}_i)$ where $\mathbf{G}_i$ is the local subnetwork composed of the neighboring nodes of a node i in such a way that the node i is not included in $\mathbf{G}_i$. Local efficiency displays the efficiency in exchanging information at a local scale around a generic node. Between 0 and 1, a higher value indicates the greater efficiency in local information exchange.

(iii) According to our modified definition, omega can be computed on the basis of the comparison of global and local efficiency between a network of interest and its equivalent extreme networks. The omega, ω, of a network of interest, **G**, is provided as the difference of two ratios: $\omega(\mathbf{G}) = E_{glob}(\mathbf{G})/E_{glob}(\mathbf{G}_{rand}) - E_{loc}(\mathbf{G})/E_{loc}(\mathbf{G}_{latt})$ where $\mathbf{G}_{rand}$ and $\mathbf{G}_{latt}$ are equivalent random and regular networks, respectively, with the same number of nodes, edges, and degree distribution as **G**. Omega represents the degree of being close to small-world topology. Between -1 and 1, a value around zero indicates small-world topology, whereas positive and negative values far from zero indicate approaches to random and regular topology, respectively.

Supplementary Tables and Figures

Table S1. Sixty gray matter regions for each pair of which white matter fiber tracts were specified to construct brain structural networks.

No	Region name
1	Hippocampus L
2	Hippocampus R
3	Amygdala L
4	Amygdala R
5	Anterior medial temporal lobe L
6	Anterior medial temporal lobe R
7	Anterior lateral temporal lobe L
8	Anterior lateral temporal lobe R
9	Ambient and parahippocampus gyri L
10	Ambient and parahippocampus gyri R
11	Superior temporal gyrus L
12	Superior temporal gyrus R
13	Inferior middle temporal gyrus L
14	Inferior middle temporal gyrus R
15	Fusiform gyrus L
16	Fusiform gyrus R
17	Cerebellum L
18	Cerebellum R
19	Insula L
20	Insula R
21	Lateral occipital lobe L
22	Lateral occipital lobe R
23	Anterior cingulate gyrus L
24	Anterior cingulate gyrus R
25	Posterior cingulate gyrus L
26	Posterior cingulate gyrus R
27	Middle frontal gyrus L
28	Middle frontal gyrus R
29	Posterior temporal lobe L
30	Posterior temporal lobe R
31	Inferior lateral parietal lobe L
32	Inferior lateral parietal lobe R
33	Caudate nucleus L
34	Caudate nucleus R
35	Accumbens nucleus L
36	Accumbens nucleus R
37	Putamen L
38	Putamen R
39	Thalamus L

40	Thalamus R
41	Pallidum L
42	Pallidum R
43	Precentral gyrus L
44	Precentral gyrus R
45	Gyrus rectus L
46	Gyrus rectus R
47	Orbito-frontal gyrus L
48	Orbito-frontal gyrus R
49	Inferior frontal gyrus L
50	Inferior frontal gyrus R
51	Superior frontal gyrus L
52	Superior frontal gyrus R
53	Postcentral gyrus L
54	Postcentral gyrus R
55	Superior parietal gyrus L
56	Superior parietal gyrus R
57	Lingual gyrus L
58	Lingual gyrus R
59	Cuneus L
60	Cuneus R

L, left; R, right.

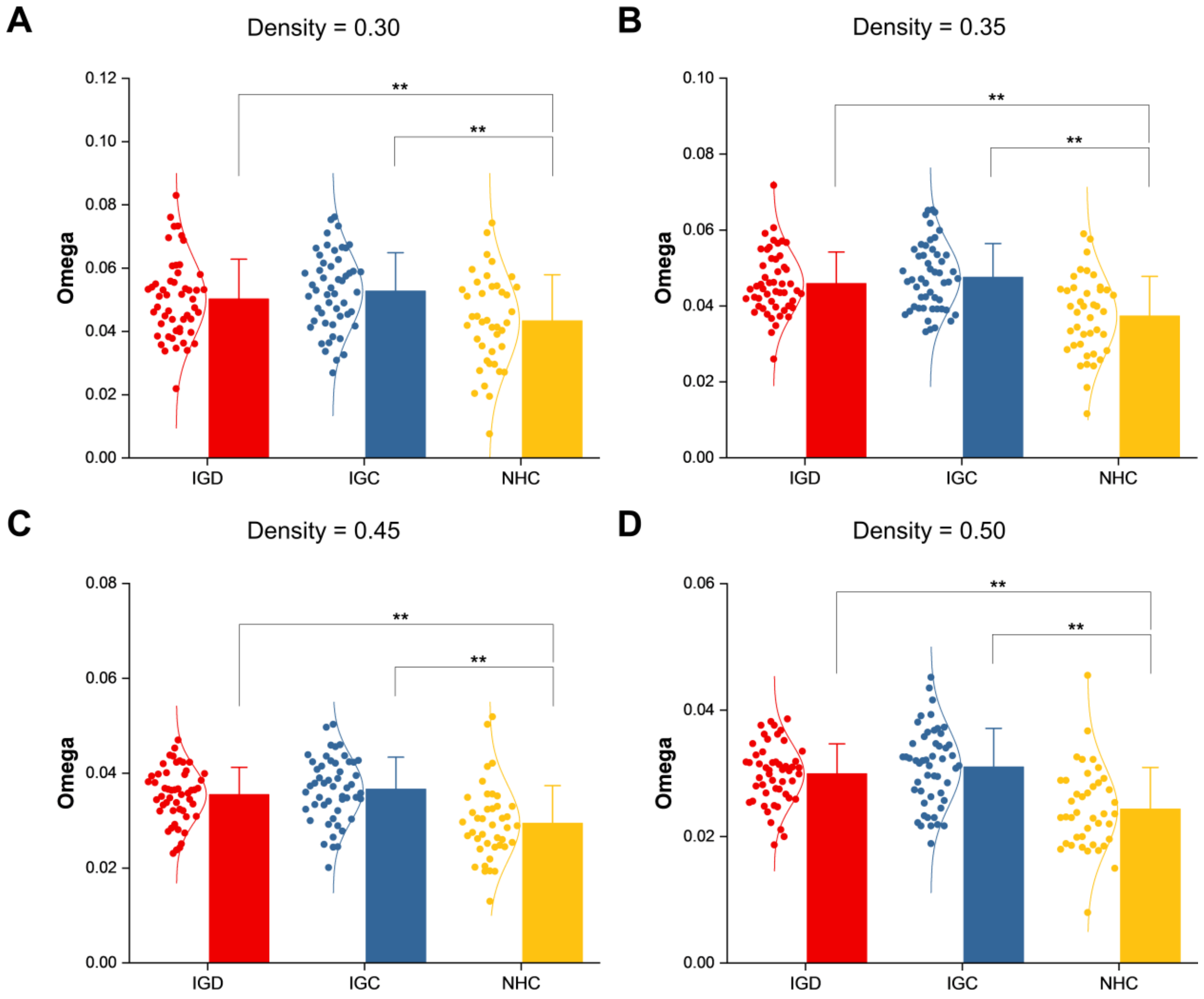

Fig. S1. Comparison of omega according to the selection of different density, 0.30 (a), 0.35 (b), 0.45 (c), and 0.50 (d), in constructing brain structural networks. The network measure was compared between gaming individuals, comprising two subdivisions labelled IGD (Internet gaming disorder) and IGC (Internet gaming control), and non-gaming healthy controls labelled NHC. Values of the network measure are represented as dots, along with a normal distribution curve fitted to them. The mean and standard deviation of the values in each group are indicated by a bar and an error bar, respectively. Double asterisks signify statistical significance corrected for multiple comparisons.

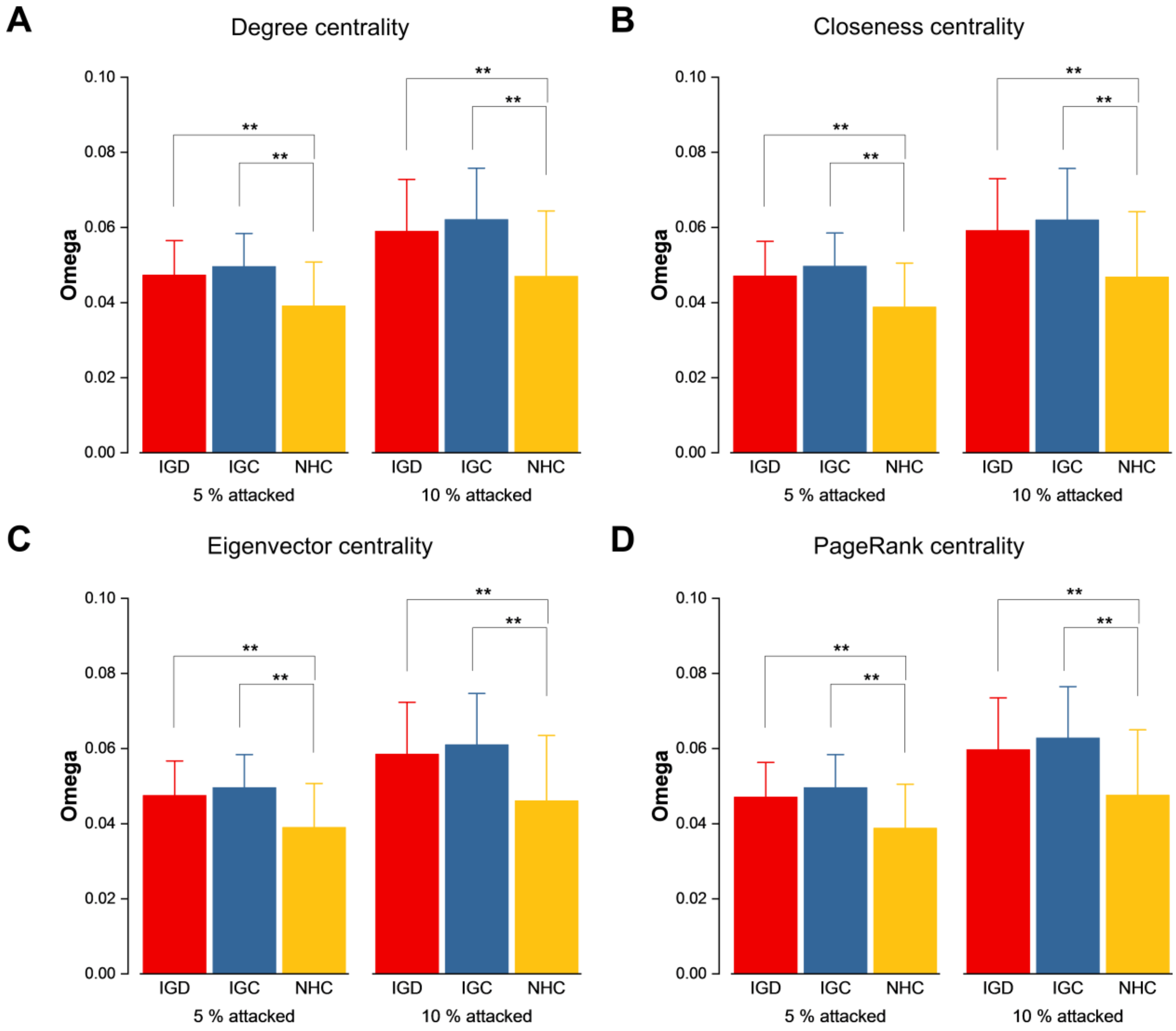

Fig. S2. Comparison of omega according to the adoption of different measures, degree centrality (a), closeness centrality (b), eigenvector centrality (c), and PageRank centrality (d), as the criteria for simulating targeted attacks on 5 % and 10 % of all nodes in brain structural networks. The network measure was compared between gaming individuals, comprising two subdivisions labelled IGD (Internet gaming disorder) and IGC (Internet gaming control), and non-gaming healthy controls labelled NHC. The mean and standard deviation of the values of the network measure in each group are indicated by a bar and an error bar, respectively. Double asterisks signify statistical significance corrected for multiple comparisons.

References

1 Latora, V. & Marchiori, M. Efficient behavior of small-world networks. *Phys. Rev. Lett.* **87**, 198701 (2001).

2 Watts, D. J. & Strogatz, S. H. Collective dynamics of 'small-world' networks. *Nature* **393**, 440-442 (1998).
